# The New York Institute of Stomatology

**Published:** 1900-11

**Authors:** 

**Affiliations:** The New York Institute of Stomatology


### Reports of Society Meetings.

### THE NEW YORK INSTITUTE OF STOMATOLOGY.

A MEETING of the Institute was held at the office of Dr. James B. Locherty, 136 West Forty-eighth Street, Tuesday evening, June 5, 1900, the President, Dr. E. A. Bogue, in the chair.

The minutes of the previous meeting were read and approved.

### COMMUNICATIONS ON THEORY AND PRACTICE.

Dr. C. F. Allan presented a communication entitled "Is Porcelain Inlay Work Impracticable?"

(For Dr. Allan's paper, see page 717.)

### DISCUSSION.

*Dr. J. H. Downes.*—Mr. Chairman, while I came here as a guest and only to listen, I have been greatly pleased with Dr. Allan's description of inlaying as it is done by Dr. Jenkins, and I would like to ask him if he can tell about the temperature at which the porcelain body fuses.

*Dr. Allan.*—I am not certain as to the fusing points.

*Dr. Downes.*—Does he take an impression of the cavity?

*Dr. Allan.*—No; he takes the matrix directly from the cavity. Dr. Jenkins prepares his cavities without undercut. With these little stones he cuts in the side of the inlay grooves for holding the cement. He depends a great deal upon the perfect fit of the inlay for support.

*Dr. F. Milton Smith.*—I would like to ask Dr. Allan whether in proximal cavities more separation would be required than in inserting a gold filling.

*Dr. Allan.*—I think it would depend a great deal upon the operator; some men require more separation than others. I generally require four thicknesses of India tape.

*Dr. Smith.*—I think it would require much more separation than I find necessary for an ordinary gold filling in an approximal cavity. I do not raise this as an objection, for if we need the space we can get it. I recently set an inlay made of gold in this manner. I feel that if I had had a little more space the result might have been better, although I am very well pleased with it. I think this inlay work has a great future and do not see so many weak points about it as have been suggested. It has been suggested that the cement is the weak point, but there are similar points to gold fillings, as some of us have found out to our sorrow. Regarding the weakness of inlays because of the cement, we have only to recall the cases of those men who for several years have been replacing the worn-down tips of teeth with soldered gold tips. These men will tell you that they consider this among the most permanent work they do. As regards large fillings in the back of the mouth, I can imagine the relief to the patient in having the work done out of the mouth. A few months ago I contoured with gold soldered inlays three teeth for a lady patient. The work done in the ordinary way with gold filling would have taken hours and the results would not have been nearly so satisfactory. Had I used porcelain I should have done much better for my patient. It would appear that the principal difficulty with this class of work is that comparatively few in the profession will be willing to master the minute details connected with its successful prosecution.

My judgment is that every man who will master the mechanical part of it so that his inlays will fit perfectly will be much pleased with the work.

*The President.*—There is one question regarding these low-fusing inlays which has not, as yet, been satisfactorily answered. I refer to the question of their durability.

*Dr. C. F. Allan.*—So far as I know they have undergone the same tests that the high-fusing inlays have been put to, and I can see no reason why they should not last.

*The President.*—I wrote to Dr. Jenkins once asking him how long he had been using these particular inlays and what their durability was, but I got no reply to this part of my letter. I think that Dr. Jenkins's inlays, as such, have been in use three or four years.

*Dr. Geo. S. Allan.*—Relative to high-fusing inlays, I called on Dr. Head, of Philadelphia, some time ago. Dr. Head is the advocate of the high-fusing inlay. I met with the most courteous reception. He showed me all the steps of his method of operating. He had a patient, a gentleman with the mesial surfaces of the first and second bicuspids gone. There was destruction of at least one-third of the teeth. It was evidently an extreme case. I saw the whole process from the making of the matrix to the baking of the porcelain. The whole operation was short of two hours, possibly not more than an hour and a half. The patient was subjected to no discomfort. These inlays after they were set were perfect imitations of nature. Although I did not examine them very carefully, to all appearance the edges, color, and contour were perfect. It is certain that in these cases, where there is only a very thin line of cement exposed, there would be nothing like the wasting away which occurs in a cavity filled with cement alone. I have been placing in inlays for some time, but I feel that I have taken hold of it wrong end foremost. I am perfectly satisfied that the process has real merits, and the man who does it successfully will please himself and his patients and do work that will not only last, but artistic work. Where I have failed was in thinking that I could take a matrix from an impression and have some one else do the baking. I believe, to succeed, one must do all the work himself. The process does away with half the labor in preparing the cavity, the edges being the main thing; the general shape of the cavity is not a matter of much importance. The insertion of the inlay is almost play for the patient. The inlay fitting the cavity satisfactorily, the operator has only to see that the cavity is kept dry and the inlay is pressed uniformly and steadily to position. A few undercuts is sufficient to hold it in position. I trust that this method will be pushed to its fullest extent in this country, and that the time will soon come when there will be many Dr. Heads doing this work. I think Dr. Jenkins deserves our commendation for having brought this process before the public.

Regarding the old method of grinding up a piece of tooth to fit the cavity, I have cases like this in my practice which have been in the mouth six or eight years, and even longer, and they show perfect to-day. In my own mouth I have three or four that Dr. Raymond put in for me three or four years ago, and they are a great source of comfort to me and as perfect to-day as when they were first inserted.

*Dr. I. F. Wardwell.*—I would like to emphasize what Dr. Allan has said regarding the absence of discomfort or pain where this method of inlays is employed. In inserting a gold filling it is generally not the preparation of the marginal walls that causes the discomfort, but the preparation of the undercuts necessary to retain the filling. I have recently employed inlays in a number of cervical cavities which were very sensitive, and had I used gold would have been more painful. I am very thankful that Dr. Jenkins has given us this idea, because I have been in the habit, in days gone by, of grinding up the inlays to fit the cavity. A lady was in the office the other day for whom I ground in one of these inlays six or eight years ago. It was in perfect condition, and she said she had had a great deal of comfort from having so conspicuous a place fixed in that manner.

*Dr. Geo. S. Allan.*—It was only last week that in a buccal cavity in a lower molar I placed an inlay with a minimum amount of pain to the patient. After thoroughly preparing the edges of the cavity I was not particular in removing all the softened dentine and giving it a retaining shape, but after rendering it aseptic I set the inlay with very little discomfort to the patient. I used nitrate of silver in the cavity during the excavating.

*The President.*—Can Dr. Allan suggest anything regarding the shadow which is said to come from the line of cement?

*Dr. C. F. Allan.*—I fancy that in these inlays where the line of cement is reduced to the minimum, the shadow effect would not be very noticeable.

*The President.*—Dr. Head and Dr. Hoffheintz have both had somewhat to say regarding shadows in inlays placed in lateral cavities. Dr. Head read a paper before the Odontological Society a few weeks ago in which he showed that inlays which absolutely matched the tooth in which they were to be inserted when viewed laterally were very much darker than the tooth. It is a condition that it would be well to watch for and look into.

*Dr. C. F. Allan.*—I might suggest that this darkening may be due to a different reflection of light. Two years ago I ground in an inlay in a cervical cavity in a central incisor for my daughter. Looked at directly in front the shade was perfect, but viewed from another direction it was not so good. I attributed it to the fact that I had polished the surface of the enamel of the inlay, causing a difference in the reflection of the light.

Charles O. Kimball, M.D., then read a paper entitled "The Dentist and his Associates."

(For Dr. Kimball's paper, see page 709.)

DISCUSSION.

*Dr. C. A. Brackett.*—Although a member for years, I believe this is the first occasion of an ordinary meeting of this organization which I have been able to attend.

Some years ago in connection with a literary organization I had relations with some public speakers. I remember the case of one honorable gentleman who was to speak upon the subject of "Shakespeare." He said with reference to the suggestion of his speaking concerning another prominent character that he would not dare to speak concerning him, because he had only been studying his life for a short time, two years, and that he did not feel competent to say anything concerning the biography of that man. He had been studying Shakespeare for thirty-five years. Our friend who has read this paper is in a similar position. Drawing from the richness of a long experience he knows whereof he speaks. In listening to him I have been immensely gratified.

I have had experience in these relations both as assistant and as principal, and in every such association the arrangements have been advantageous for the principal, for the assistant, and for the people served. I cannot conceive how the subject could have been more ably or profitably treated than it has been by the essayist. The relationship as described seems ideal. I am much gratified with what he said concerning the qualities which are desirable in an associate; that in addition to a high order of professional ability he should have a firm grounding in excellent moral principles; that he should be a chaste man, a man whose amusements are of a wholesome and ennobling kind, and, above all, that he should be a man having a genuine respect for noble womanhood. All of these things impressed me, as I know they impressed all here present, very forcibly.

One other thought. It has been my privilege for many years to see many young men in our dental schools and recently graduated, and I have wished over and over again that they had a better appreciation of the opportunities that are before them in the world, and of the great mountain of work that is waiting to be done by those who make themselves competent personally, professionally, and morally. I have always found it difficult to sympathize with a man who cannot find work to do. I most earnestly wish that the students in our colleges could have a better appreciation of how the older men are watching them, looking for able assistance as their own work increases and their powers fail. I wish all young men might be more profoundly impressed with the importance of the diligent use of time, with the value and the limitations of life, and with a full sense of the opportunities for achievement which lie before them.

*Dr. C. F. Allan.*—The paper is so excellent, the thoughts so well expressed, that I shall not attempt to discuss it, but shall wait until I can read it and ponder over and try and remember and profit by some of the beautiful thoughts.

*Dr. A. L. Swift.*—I have nothing to say in discussion. But wish to express my appreciation and thanks to the essayist for his most excellent paper.

*Dr. E. S. Robinson.*—I have listened with more than ordinary interest to this paper, as it has been my privilege to have been associated with Dr. Kimball both as a student and as an associate for eight years. I am happy to say that the training I received under him will be of the greatest value and service to me throughout my professional life.

*Dr. Geo. S. Allan.*—I regret to say that I never had any training in my younger days under a man whom I can look back upon with respect, and so had to work out my own salvation as best I could, but I can appreciate how fortunate a young man is who is able to get into an office and receive the training which Dr. Kimball mentions. I am very glad that I had the privilege of listening to this paper.

*Dr. J. B. Locherty.*—My sentiments are certainly fully in accord with those of Dr. Kimball. I had the pleasure of being associated in the most cordial manner, during the earlier part of my professional life, with a man who is a guest of the Institute to-night, Dr. Downes, and the benefit to be derived by the associate from an excellent preceptor is of inestimable value in fitting the young practitioner for a future professional career.

*Dr. W. M. Whitlock.*—I hardly feel competent to discuss this paper. The field properly belongs to men much older and longer in practice than myself. I think, however, that something may properly be said from the stand-point of an associate, and from the associate's point of view I venture a word of approval and appreciation of the essay.

It does not seem to me that there is a word or a thought in the paper that a properly constituted young man could take exception to. The relation between the principal and associate mentioned by the essayist is ideal. While there is and ought to be financial benefit accruing to the principal under this arrangement, the benefits received by the associate in the way of instruction and introduction are so great that it is he who receives the greatest gain. I feel that if, when I left college, I had started in practice as an assistant without further training, I would have fallen into a rut that I could never have gotten out of, and the services given my patients would not have compared with those given after the careful training spoken of by Dr. Kimball. The value of being able to consult at any time one who has had experience cannot be overestimated. It is certain that the incentive to the associate who is building up a practice and having the benefits of the experience of men who have had years of practice is much greater and more ennobling than it can be to the assistant who is waiting for Saturday night to draw his salary.

Dr. Kimball used a very beautiful symbol, "the passing of the torch." To pass a torch it is necessary to have a torch to pass; in other words, to train an associate it is necessary to have something definite to give him. The principal should have, rooted to his very life, an ideal which years of experience have convinced him is the best, and he should insist upon the associate becoming master of it. His life and work should be of such a high standard that it will be an inspiration and a goal towards which the younger man will work. I can say with all earnestness, as an associate, that this has been my experience. I shall ever be grateful that at the beginning of my professional life I have had the benefit, through association, of experience and knowledge accumulated through generations.

*Dr. S. E. Davenport.*—It is usually the theorist who has the most clearly defined ideas and the best way of expressing them, but we have listened to-night to an essay upon a chosen subject by a gentleman who is a practical man in the field and one with long experience in the training of associates. We have also heard the expressions of confidence and approval from several of the brilliant results of this training. We certainly are all under obligation to Dr. Kimball for his paper, which shows such careful preparation. There is, I know, a great difference of opinion as to which is the best plan of relationship between principal and associate, but I think we have been shown by Dr. Kimball, as the result of his long experience, the way in which both parties to the contract will be best served. There are, of course, difficulties in the way of making an essay complete on this subject in all its practical bearings, because it is necessary to go over such a large field, and especially in this instance the Executive Committee requested the essayist to condense his remarks into a shorter period of time than he really should have had. It is for this reason, no doubt, that we are left in the dark regarding so many points concerning which it would be interesting to have Dr. Kimball give us further information.

*Dr. J. Morgan Howe.*—I cannot let the opportunity pass without congratulating Dr. Kimball upon his paper; I wish to thank him very much. It has been suggested that the great advantages of the association he has discussed are that the associate is able to give to his patients much better services than he would without such pupilage, and also that he is able to establish a practice among a desirable class of patients in a much shorter time than he otherwise could, thus starting his professional life earlier. In that regard it is comparable, I think, to the experiences of young men in the other professions, where it is becoming more and more necessary for them to become associated with practitioners of established reputation in order to have a reasonable chance of success. It occurs to me that this may be one of the signs of our professional development.

*Dr. F. Milton Smith.*—I think it has never been my privilege to listen to a paper on any subject in any dental society that has more forcibly or pleasantly impressed me. I presume the paper impressed me more because I am one of those unfortunate men who, after graduating from the dental college, never had the advantages of association with such a man as Dr. Kimball. I can say without hesitation that I would have gladly given five years after graduating from college, without a penny of compensation, if those five years could have been spent under the careful training of such a man, for then I should have had a foundation which I think something might have been built upon. As it is, I have had to learn by hard knocks what little I have been able to learn in dentistry.

*The President.*—The gentlemen here, I think, will all agree with me that we owe our thanks to Dr. Kimball for his carefully prepared paper and on a subject that apparently had little to recommend it. I hope he will favor us with a full answer to those who have discussed his paper.

*Dr. Kimball.*—There is very little to say excepting this, that it is impossible in going over so much ground in a paper to give full details. I shall be very glad at any time to answer any questions regarding the methods and minute workings out of such a plan. As I stated in my paper, I have observed the working of this plan, not only in my own office but in the office of Dr. Dunning and his successors, and also in the office of quite a number of my friends. Without exception, where these principles have been carried out, where the associate has been carefully selected, and where the principal himself has had something to give, the results have been most satisfactory. Of course the principal must have something to give. You cannot get something from nothing, and unless he has a real professional character and is willing and anxious to impart his professional experience to his associate, the plan cannot succeed. In these young men we see growing up the men who are going to make the strong, helpful, and efficient men in the years that are to come, and I feel fully persuaded that in this way alone can we hope to make our profession what it should be. Any idea of the profession gaining in character by outside influence, that is to say, by dinners or by shows of any kind, is altogether false. If we are to be a profession looked up to and respected by other professions we must be ourselves men whose character is based on broad and deep foundations built up by liberal instruction. We must be such men as we see in the ranks of our leading clergymen, lawyers, and physicians. You know what they are. They are almost all of them college-bred men. They have had broad advantages. We must see to it that our young men also have these advantages; this is the only way in which the profession can ultimately stand on a par with the other professions. I believe if we all of us were as thoroughly rooted and grounded in general training as we are in our professional specialty, we should exert a broader influence than we do. I say this very plainly because I am among my friends whom I love and respect, but there are many men in the profession to-day who would be stronger and more helpful men if they had had better advantages when they started. Think how much fuller some of our lives would have been if, in addition to all we have been able to acquire, we had had a broader and a deeper foundation upon which to build, and let us use every influence and power we possess to see that those coming after us are so prepared.

Upon motion a vote of thanks was extended to Dr. Kimball for his excellent paper.

Adjourned.

FRED. L. BOGUE, M.D., D.D.S.,
*Editor The New York Institute of Stomatology.*

